# Inhibitory effects of algal polysaccharide extract from *Cladophora* spp. against herpes simplex virus infection

**DOI:** 10.1038/s41598-024-60941-7

**Published:** 2024-05-24

**Authors:** Pitchayuth Srisai, Sureeporn Suriyaprom, Aussara Panya, Jeeraporn Pekkoh, Yingmanee Tragoolpua

**Affiliations:** 1 Department of Biology, Faculty of Science, Chiang Mai University, Chiang Mai, 50200 Thailand; 2 Office of Research Administration, Chiang Mai University, Chiang Mai, 50200 Thailand; 3 Natural Extracts and Innovative Products for Alternative Healthcare Research Group, Faculty of Science, Chiang Mai University, Chiang Mai, 50200 Thailand

**Keywords:** Herpes virus, Pharmaceutics, Outcomes research

## Abstract

Herpes simplex virus (HSV) is a causative agent of fever blister, genital herpes, and neonatal herpes. Nowadays, edible algae are recognized as health food due to high nutrition content and their many active compounds that are beneficial to health. The purpose of this study is to investigate the inhibitory effects of algal polysaccharide extract from *Cladophora* spp. against herpes simplex virus type 1 and type 2 on Vero cells. In this study, the structure of polysaccharide extract is presented as S=O and C–O–S of the sulfate group, as identified by the FT-IR technique. The toxicity of algal polysaccharide extract on Vero cells was determined by MTT assay. The algal extract showed low toxicity on the cells, with 50% cytotoxic concentration (CC_50_) value greater than 5000 µg mL^−1^. The inhibition of HSV infection by the algal extract was then evaluated on Vero cells using plaque reduction assay. The 50% effective concentration (EC_50_) values of algal extract exhibited antiviral activity against HSV-1 upon treatment before, during, and after viral adsorption with and without removal of the extract were 70.31, 15.17, > 5000 and 9.78 µg mL^−1^, respectively. Additionally, the EC_50_ values of algal extract against HSV-2 upon treatment before, during and after viral adsorption with, and without removal of the extract were 5.85, 2.57, > 5000 and 26.96 µg mL^−1^, respectively. Moreover, the algal extract demonstrated direct inactivation of HSV-1 and HSV-2 virions as well as inhibitory effect against HSV replication. Accordingly, algal polysaccharide extract containing sulfated polysaccharides showed strong activity against HSV. Therefore, it is proved to be useful to apply *Cladophora* spp. polysaccharide extract as an anti-HSV agent.

## Introduction

Herpes simplex is caused by herpes simplex virus (HSV) infection, particularly HSV-1 and HSV-2. The herpes simplex virus is categorized as a group I double-stranded DNA virus and is a member of the *Herpesviridae* family^1,2^. HSV-1 and HSV-2 are pervasive human pathogens that cause localized skin infections. An HSV-1 infection can cause herpes labialis, whereas an HSV-2 infection can cause herpes genitalist^3,4^. The viruses remain a prominent problem in human public health because of their high transmission rate between people. HSV-1 is transmitted primarily through direct human skin contact, whereas HSV-2 is transmitted through sexual contact involving exposure to the mucous membrane within lesions or infections, or from mucosal secretion. Moreover, HSV-2 can be transmitted horizontally and vertically during asymptomatic shedding, and is incurable, causing a latent infection in the ganglia^5–8^.

The nucleoside analogues, especially acyclovir and related analogues, e.g., valacyclovir, penciclovir, and famciclovir, were tested as standard remedies for HSV infection^9^. The main action mode of nucleoside analogues is targeting the viral DNA polymerase. However, nucleoside drug analogues continue to have side effects in the case of long-term usage and the virus may develop a drug resistance. Drug-resistant HSV strains are caused by mutations in viral thymidine kinase and viral DNA polymerase genes, and the mutant viruses do not respond to commercial drug treatment^10–12^. Moreover, the epidemiology of HSV and drug resistant HSV have increased^13^. Nowadays, there is a lot of interest in natural substances as remedies for many ailments, including herpes simplex virus infection.

Several biologically active compounds exhibit antiviral activity, such as polysaccharides, peptides, proteins, phenolic compounds, and other organic compounds^14,15^. The efficacy of biologically active compounds against viruses indicates their broad antiviral activities on different pathways of the viral multiplication cycle. Antiviral activity is observed on the viral entry, penetration, replication, assembly, and egression^16,17^. The previous reports indicated that there has been extensive research on the anti-HSV properties of various natural substances. For instance, terpenes isolated from *Melia azedarach* showed high antiviral activity against HSV-1 in cell culture experiments^18^. Additionally, β-orcinol depsidone derived from lichen *Usnea fruticose* was found to inactivate HSV-1 DNA-polymerase during HSV replication^19^. Moreover, catechin purified from *Limonium sinense* exhibited greater antiviral activity than ACV by reducing the expression of *ICP0* and *ICP4* genes. Polyphenols isolated from tea plants such as *Camellia sinensis* could interfere the fusion process between the viral and cellular membranes by aggregating HSV glycoproteins B and D on the viral surface^20,21^. Furthermore, the griffithsin (GRFT) peptide isolated from red algae directly effect on the viral glycoproteins B, D, and heterodimers of gH/gL, which are essential for virus entry and cell-to-cell spread of HSV^22,23^. Crude aqueous and organic solvent extracts from algae, including green algae (*Chlorella vulgaris* and *Spirogyra neglecta*), brown algae (*Durvillaea antarc*), and red algae (*Hypnea musciformis*), demonstrated potent anti-HSV activities^24,25^. Polysaccharides, especially sulfated polysaccharides, have high potential antiviral activity against infections during the viral adsorption to the host cell. Moreover, biological and synthetic sulfated polyanions are able to inhibit the replication of various mammalian viruses^26,27^. Sulfated polysaccharides are found in some microorganisms, plants, and animals; however, the highest level is found mostly in algae. Sulfated glucan, sulfated galactan, and sulfated arabinogalactan are the main sulfated polysaccharides found in green macroalgae^28,29^. Marine algae are also sources of various structures of sulfated polysaccharides varying with the algal species. The major sulfated polysaccharides found in marine algae include ulvan of green algae (Chlorophyceae), fucoidan and laminarans of brown algae (Phaeophyceae), and carrageenan of red algae (Rhodophyceae)^30^. Moreover, sulfated polysaccharides display several physiochemical and biological features of potential interest for food, agricultural, and pharmaceutical applications. Furthermore, sulfated polysaccharides demonstrate additional properties including anticoagulant, antiviral, antioxidant, anticancer, and immunomodulating activity^31–33^.

Therefore, the purpose of research is to study the characteristics of polysaccharide extract from *Cladophora* spp. and investigate the inhibitory effects of algal polysaccharide extract against herpes simplex virus infections.

## Results

### Algal polysaccharide extract

The extraction yield and chemical composition including total carbohydrate, protein, and sulfate content of crude *Cladophora* spp. algal polysaccharide extract from *Cladophora* spp. is shown in Table 1, as taken from this study. The extraction yield for crude polysaccharide extract was approximately 30.10% w w^−1^. The algal polysaccharide extract consisted of 51.37% w w^−1^ carbohydrate, 13.69% w w^−1^ protein, and 7.31% w w^−1^ sulfate.

**Table 1:** Yield and chemical composition of algal polysaccharide extract from *Cladophora* spp.

Chemical content (% w w^−1^)	Yield	Total carbohydrate	Protein	Sulfate
	30.10	51.37	13.69	7.31

**Figure 1:**
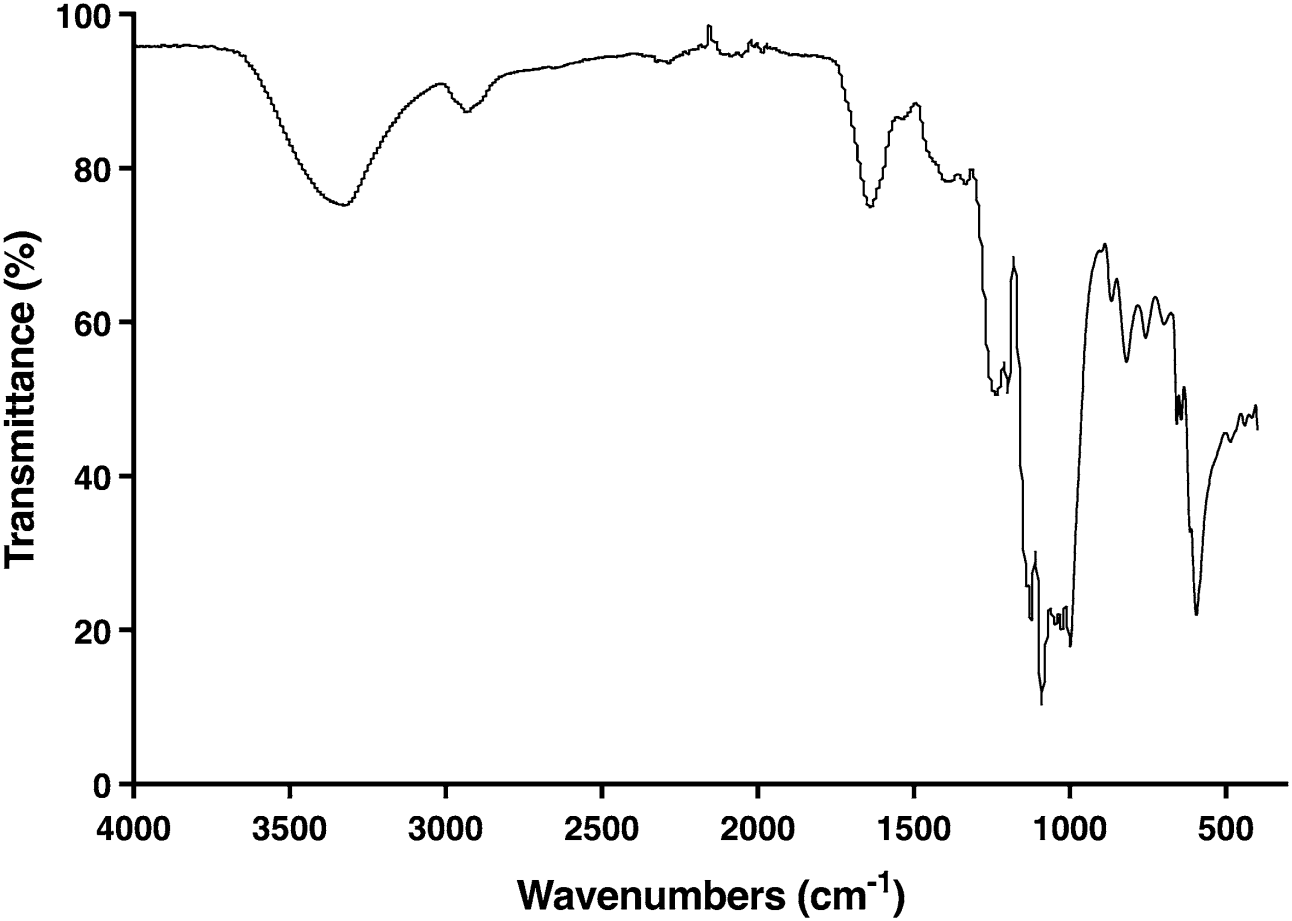
Infrared transmittance spectrum of algal polysaccharide extract from *Cladophora* spp.

The FT-IR spectrum of polysaccharide extract from *Cladophora* spp. is presented in Fig. 1. A characteristic band at 3321.1 cm^−1^ corresponds to the –OH stretching vibrations of the hydroxy groups and the N–H stretching vibrations of the amide group. A small band at 2931.6 cm^−1^ is attributed to the –CH_2_ or –CH_3_ stretching vibrations of the alkyl group, or the aliphatic hydrocarbon chain. Strong transmission at 1639.7 cm^−1^ indicates the C = O asymmetric stretching vibrations of the amide I group, and also implicates the N–H bending vibrations of the amide II group, indicating the presence of amino acid. The 1124.3 cm^−1^ peak represents the C–O stretching vibrations of the polysaccharide ether. Moreover, the signal at 1025.9 cm^−1^ links to the stretching vibrations of the C–O–C bridge of the glucosides and sugar ring. Specifically, four bands at 1333.5, 1235.5, 866.4, and 594.1 cm^−1^ are consistent with the S=O stretching vibrations of the sulfonamides group, the S=O asymmetric stretching vibrations of the sulfated ester substitutions, the C–O–S bending vibrations of the sulfate group and the C–S stretching vibrations of the sulfide ester substitutions, respectively. These results confirm the presence of sulfate groups (7.31%) in the polysaccharide structure.

### Cytotoxicity of algal polysaccharide extract

**Figure 2:**
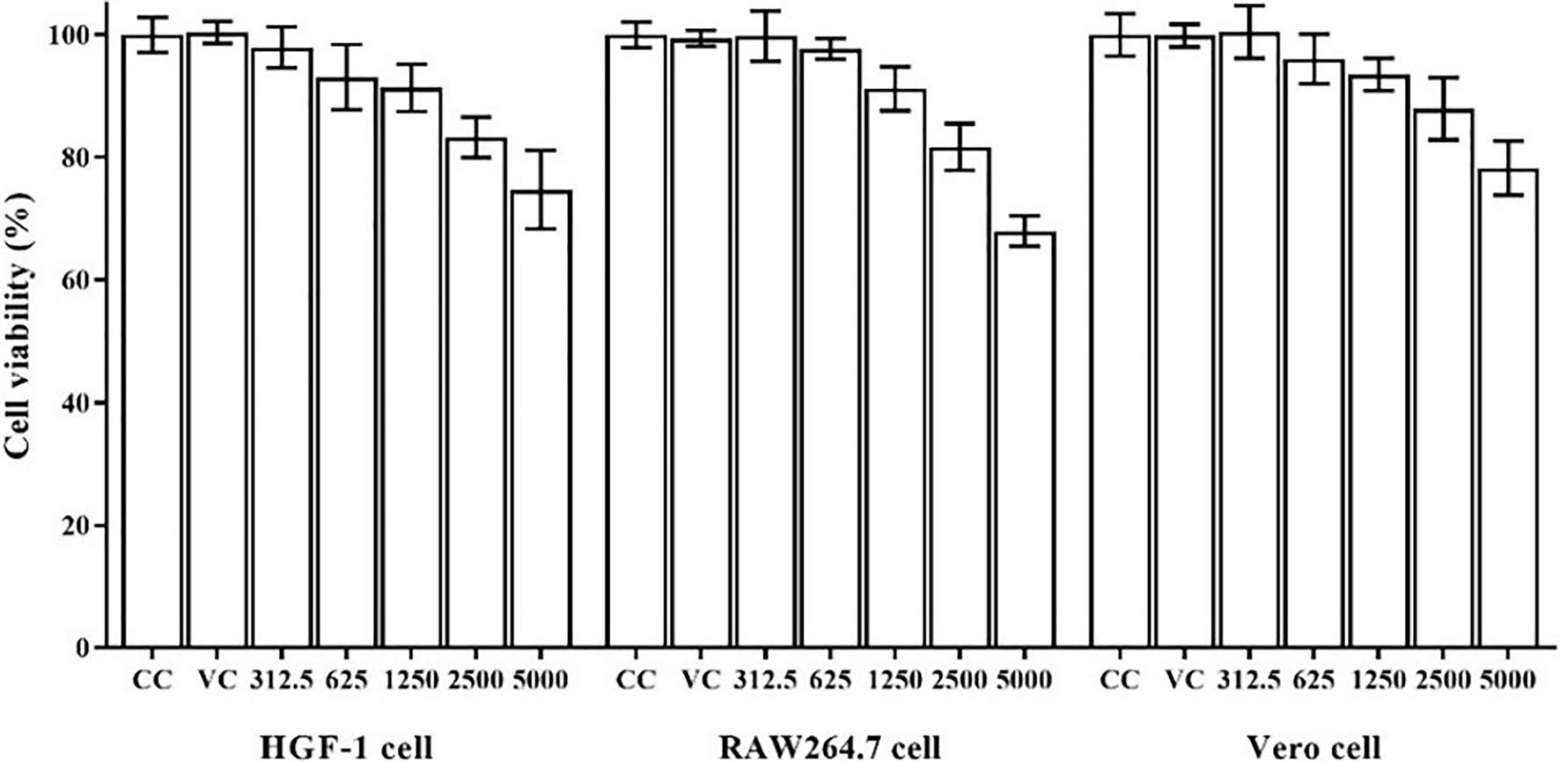
Cytotoxic effects of algal polysaccharide extract from *Cladophora* spp. on HGF-1 cells, RAW264.7 cells and Vero cells. Bar graph and error bars are based on mean ± SD of three experiments.

The cytotoxicity test of algal polysaccharide extract from *Cladophora* spp. was performed in vitro to detect cytotoxic activity on a normal human primary cell line (HGF-1 cells), immune cells (RAW264.7 cells) and a viral susceptible cell line (Vero cells). The stock solution of the algal polysaccharide extract was prepared at a concentration of 50 mg mL^−1^ by dissolving it with deionized water. The stock extract was diluted by two-fold dilution with DMEM growth medium at extract concentrations of 312.5, 625, 1250, 2500, and 5000 µg mL^−1^. The DMEM growth medium was also used as the basal media for the cell control (CC) and sterile distilled water was used as the vehicle control (VC). The results obtained are shown in Fig. 2. Algal polysaccharide extract had a low toxicity on HGF-1 cells, RAW264.7 cells and Vero cells. The 50% cytotoxic concentration (CC_50_) value of algal polysaccharide extract used to treat all cell lines was greater than 5000 µg mL^−1^, therefore, extract with a maximal concentration of 5000 µg mL^−1^ was used to determine the anti-HSV activity, viral particle inactivation, and antiviral replication kinetics.

### Anti-viral activities of algal polysaccharide extract

**Figure 3:**
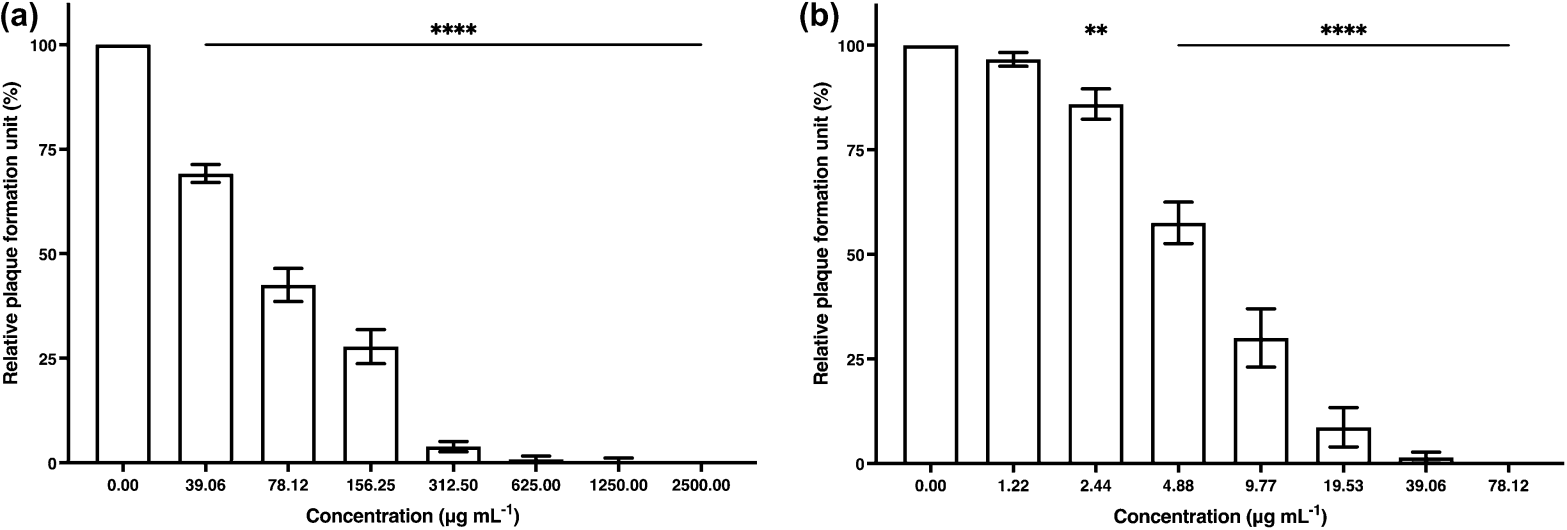
The plaque reduction of (**a**) HSV-1 and (**b**) HSV-2 activity by algal polysaccharide extract from *Cladophora* spp. upon treatment before viral adsorption on Vero cells. Graphs and error bars are based on mean ± SD of three experiments. *p < 0.05, **p < 0.01, ***p < 0.001, ****p < 0.0001.

The antiviral activity of algal polysaccharide extract from *Cladophora* spp. against HSV-1 and HSV-2 was evaluated by plaque reduction assay. The Vero cells and the HSV-infected Vero cells were treated with different concentrations of algal polysaccharide extract at a maximal concentration of 5000 μg mL^−1^. Algal polysaccharide extract at concentrations of 78.12 and 1250 μg mL^−1^ showed efficient inhibition of HSV-1 and HSV-2 upon treatment before viral adsorption to the Vero cells, with both percentages of inhibition at 100% (Fig. 3). Heparin at concentration of 20 mg mL^−1^ was used as a positive control to inhibit HSV-1 and HSV-2 upon treatment before viral adsorption to the Vero cells, with the percentage of inhibition of 13.15 and 10.61%, respectively.

**Figure 4:**
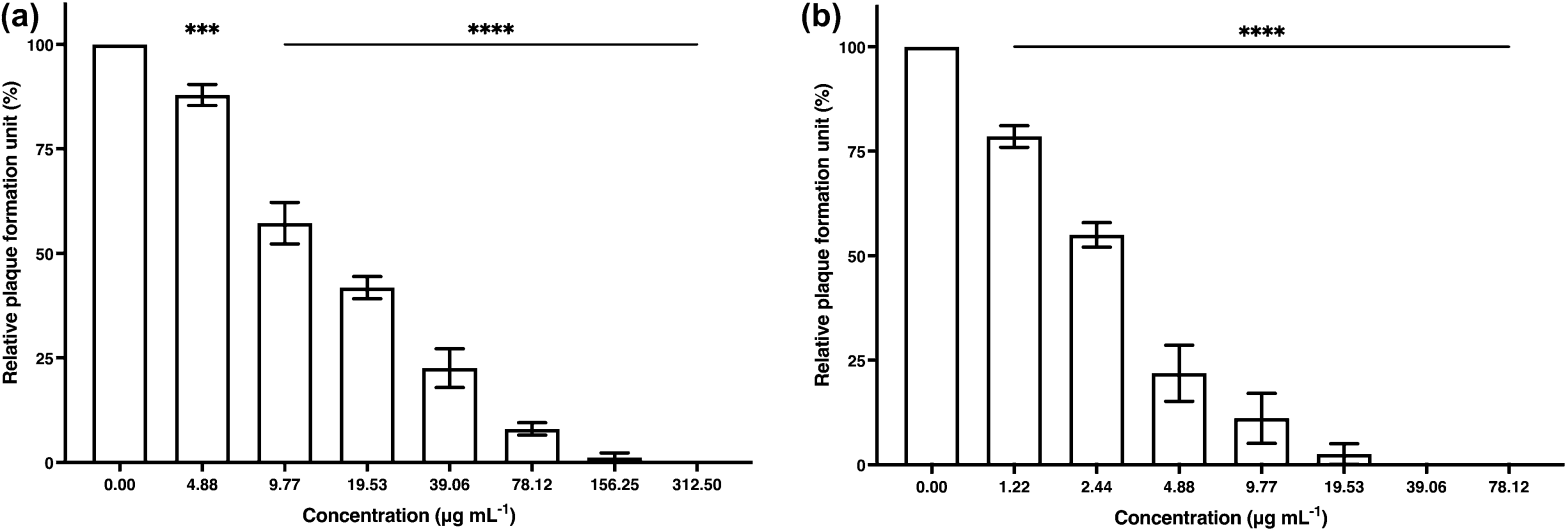
The plaque reduction of (**a**) HSV-1 and (**b**) HSV-2 activity by algal polysaccharide extract from *Cladophora* spp. upon treatment during viral adsorption on Vero cells. Bar graph and error bars are based on mean ± SD of three experiments. *p < 0.05, **p < 0.01, ***p < 0.001, ****p < 0.0001.

Additionally, algal polysaccharide extract at concentrations of 39.06 and 312.50 μg mL^−1^ showed potential to eliminate HSV-1 and HSV-2 upon treatment during viral adsorption to the Vero cells, with both percentages of inhibition at 100% (Fig. 4). Heparin at a concentration of 20 mg mL^−1^ was also used as a positive control to inhibit HSV-1 and HSV-2 upon treatment during viral adsorption to the Vero cells, with the percentage of inhibition at 97.23 and 86.59%, respectively.

**Figure 5:**
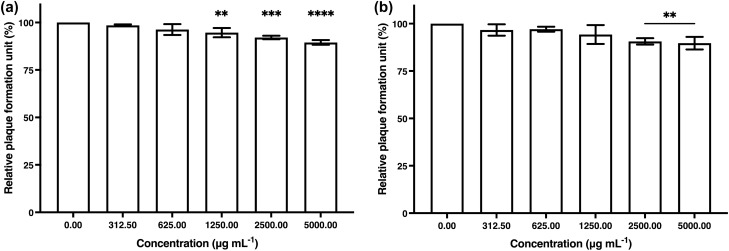
The plaque reduction of (**a**) HSV-1 and (**b**) HSV-2 activity by algal polysaccharide extract from *Cladophora* spp. upon treatment after viral adsorption on Vero cells with removal of the extract after treatment. Bar graph and error bars are based on mean ± SD of three experiments. *p < 0.05, **p < 0.01, ***p < 0.001, ****p < 0.0001.

The treated cells were incubated at room temperature for 1 h. After incubation, the algal polysaccharide extract was removed, and the treated cells were washed twice with phosphate-buffered saline solution to determine the single cycle of HSV-2 infection. The resulted showed that algal polysaccharide extract at the highest concentration of 5000 μg mL^−1^ demonstrated low percentage of inhibition by 20% (Fig. 5). Moreover, ACV at a concentration of 10 μg mL^−1^ was also used as a positive control to inhibit HSV-1 and HSV-2 viral infection on Vero cell, with the percentage of inhibition at 90.54 and 31.88%, respectively.

**Figure 6:**
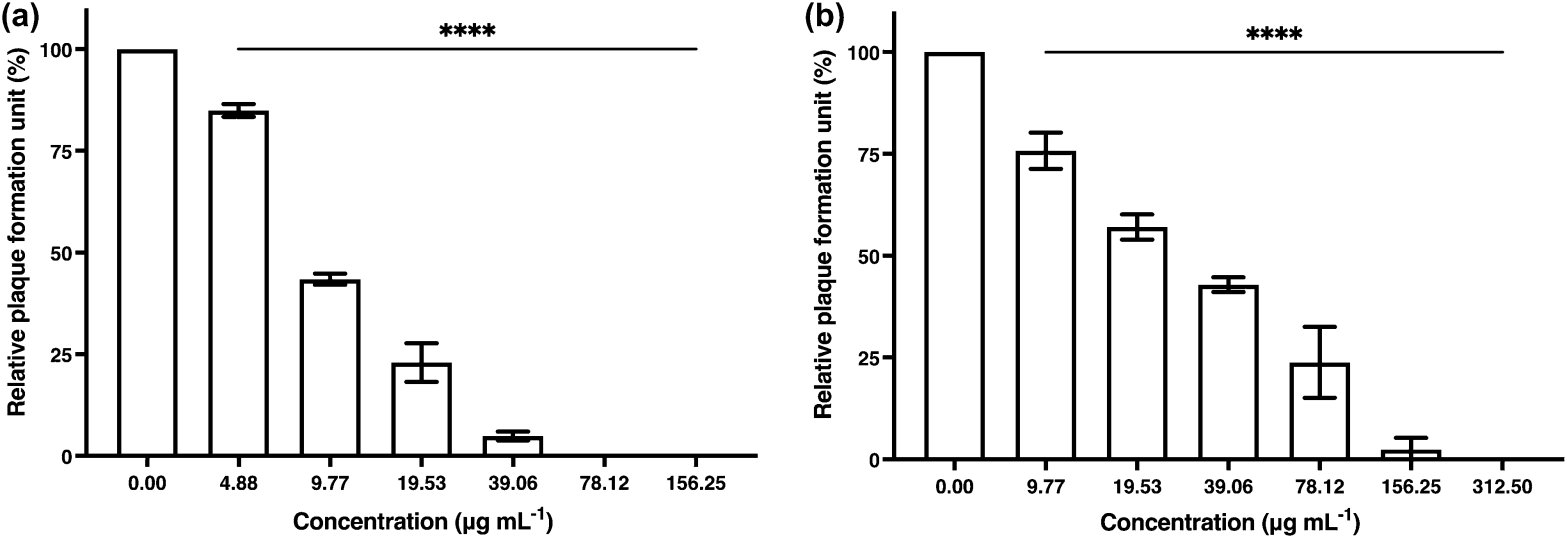
The plaque reduction of (**a**) HSV-1 and (**b**) HSV-2 activity by algal polysaccharide extract from *Cladophora* spp. upon treatment after viral adsorption on Vero cells without removal of the extract after treatment. Bar graph and error bars are based on mean ± SD of three experiments. *p < 0.05, **p < 0.01, ***p < 0.001, ****p < 0.0001.

Furthermore, algal polysaccharide extract at concentrations of 156.25 and 312.50 μg mL^−1^ also showed high potential to eradicate HSV-1 and HSV-2 by 100% upon treatment after viral adsorption without removal of the algal polysaccharide extract (Fig. 6). ACV at a concentration of 10 μg mL^−1^ was used as a positive control to inhibit HSV-1 and HSV-2 upon treatment after viral adsorption to the Vero cell, with the percentage of inhibition at 97.64 and 49.25%, respectively.

The 50% effective concentration (EC_50_) values of algal polysaccharide extract from *Cladophora* spp. against HSV-1 upon treatment before, during and after viral adsorption with, and without removal of the extract were 70.31, 15.17, > 5000 and 9.78 µg mL^−1^, respectively. In addition, the EC_50_ values of algal polysaccharide extract from *Cladophora* spp. against HSV-2 when treated before, during and after viral adsorption with, and without removal of the extract were 5.85, 2.57, > 5000 and 26.96 µg mL^−1^, respectively (Table 2).

**Table 2:** Inhibition of HSV by algal polysaccharide extract from *Cladophora* spp. upon treatment before, during and after viral adsorption with and without removal the algal extract from the infected Vero cells.

Inhibition mechanisms		HSV-1	HSV-2
Before viral adsorption	EC_50_ (µg mL^−1^)	70.31	5.85
	Selective Index (SI)*	> 71.11	> 854.70
During viral adsorption	EC_50_ (µg mL^−1^)	15.17	2.57
	Selective Index (SI) *	> 329.60	> 1945.52
After viral adsorption with removal of the algal extract	EC_50_ (µg mL^−1^)	> 5000	> 5000
	Selective Index (SI)*	1	1
After viral adsorption without removal of the algal extract	EC_50_ (µg mL^−1^)	9.78	26.96
	Selective Index (SI) *	> 511.25	> 185.46

*Selective Index (SI) = 50% Cytotoxic dose (CD_50_)/50% Effective dose (ED_50_).

Furthermore, the selectivity index (SI) values of algal polysaccharide extract from *Cladophora* spp. were calculated from 50% cytotoxic dose (CD_50_)/50% effective dose (ED_50_). As 50% Cytotoxic dose (CD_50_) of the algal polysaccharide extract from *Cladophora* spp. was more than 5000 ug mL^−1^. Thus, SI values of algal polysaccharide extract from *Cladophora* spp. against HSV-1 upon treatment before, during and after viral adsorption with, and without removal of the extract were more than 71.11, 329.60, 1.00 and 511.25, respectively. Additionally, the SI values of the *Cladophora* spp. polysaccharide extract against HSV-2 upon treatment before, during and after viral adsorption with, and without removal of the extract were more than 854.70, 1945.52, 1.00 and 185.46, respectively (Table 2).

**Figure 7:**
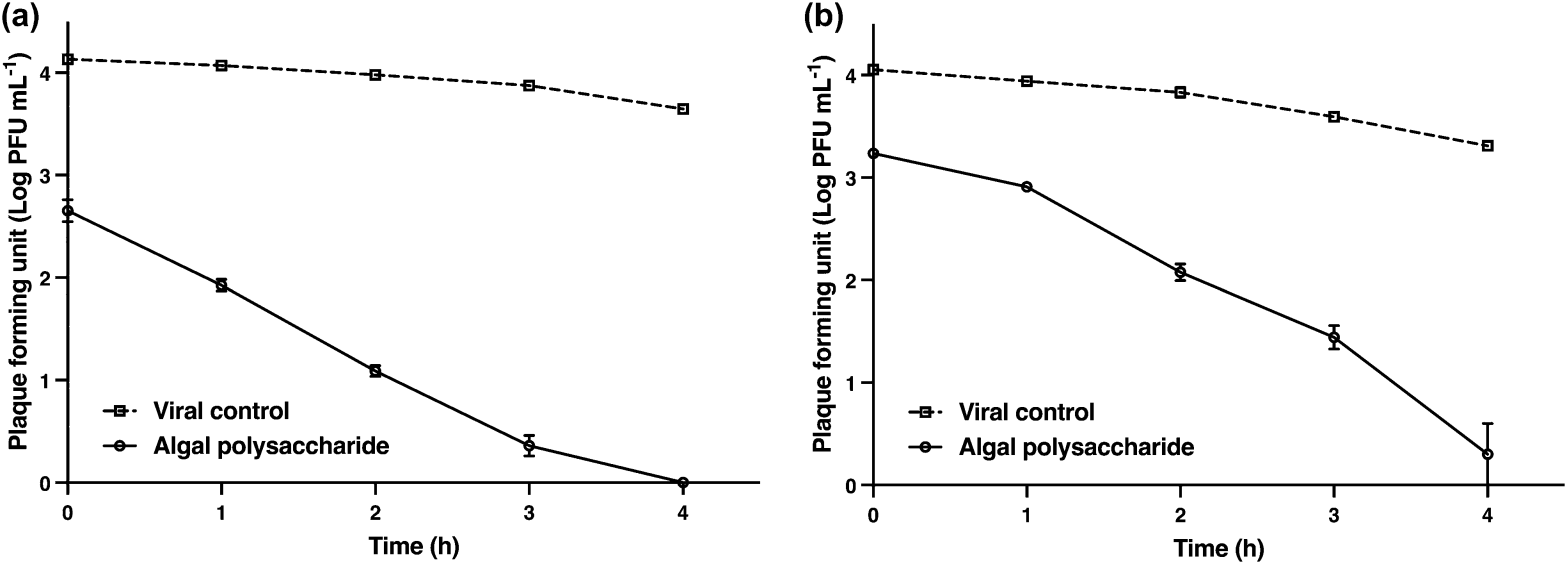
The virucidal activities of algal polysaccharide extract from *Cladophora* spp. against (**a**) HSV-1 and (**b**) HSV-2 particles in comparison with the virus control. Bar graph and error bars are based on mean ± SD of three experiments.

**Figure 8:**
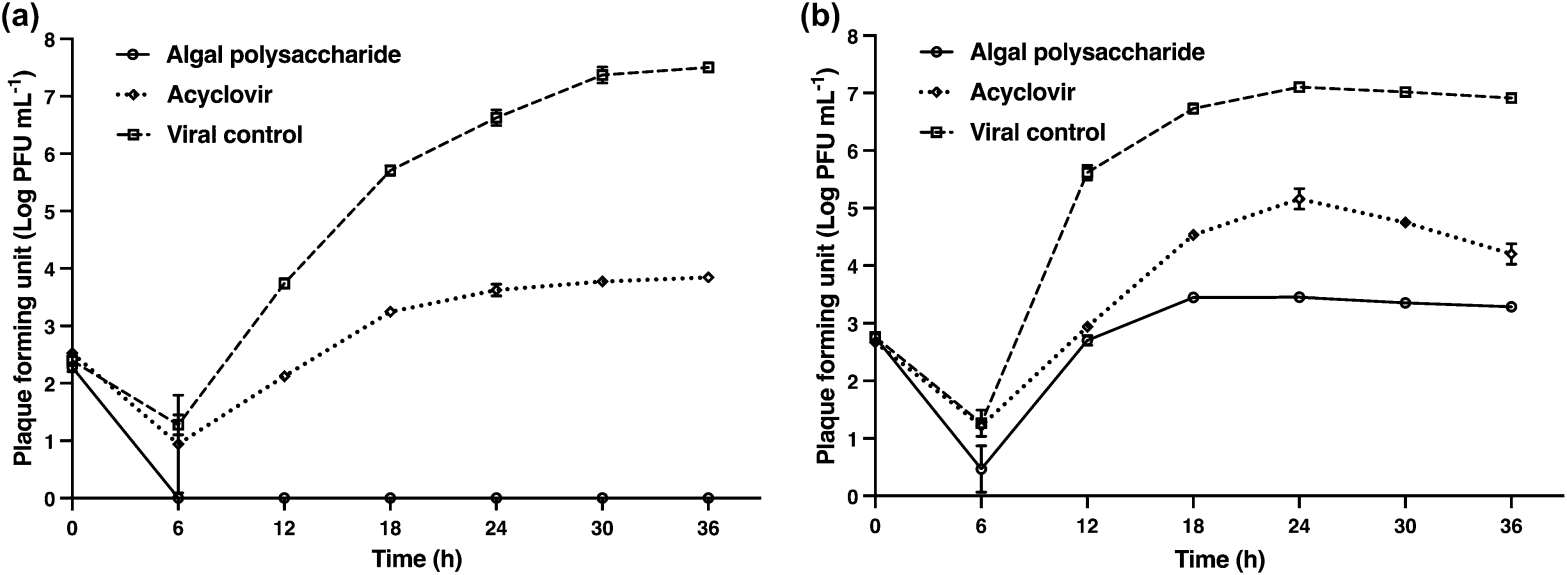
The inhibition of (**a**) extracellular HSV-1 yield and (**b**) intracellular HSV-1 yield upon treatment with algal polysaccharide extracts from *Cladophora* spp. for 0, 6, 12, 18, 24, 30, and 36 h, compared to the ACV as a positive control and viral control. Bar graph and error bars are based on mean ± SD of three experiments.

**Figure 9:**
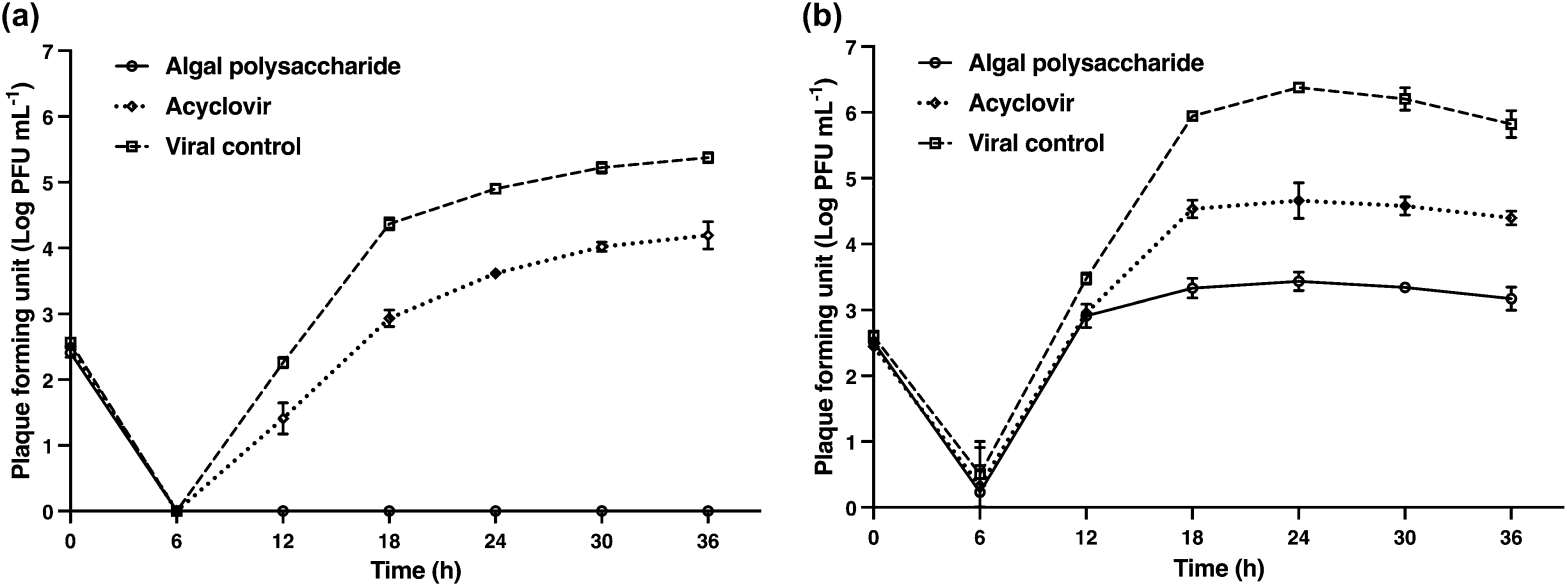
The inhibition of (**a**) extracellular HSV-2 yield and (**b**) intracellular HSV-2 yield upon treatment with algal polysaccharide extracts from *Cladophora* spp. for 0, 6, 12, 18, 24, 30, and 36 h, compared to the ACV as a positive control and viral control. Bar graph and error bars are based on mean ± SD of three experiments.

The activity of algal polysaccharide extract on the direct inactivation of HSV particles was determined using plaque titration assay and was compared with the virus control. HSV-1 and HSV-2 particles at titers of 1.0 × 10^4^ PFU mL^−1^ were treated with algal polysaccharide extract at a concentration of 5000 μg mL^−1^ for 1, 2, 3, and 4 h. The inhibitory effect of the extract on HSV viral particles was observed to be the highest when the incubation period increased (Fig. 7). The results signify that algal polysaccharide extract at a concentration of 5000 µg mL^−1^ could reduce the plaque number of HSV-1 and HSV-2 after treatment greater than 2 log PFU mL^−1^ when incubation for 1 and 3 h, respectively. Algal polysaccharide extract reduced the plaque number of HSV-1 by 1.47, 2.14, 2.89, 3.49, and 3.65 log PFU mL^−1^ when treated at 0, 1, 2, 3, and 4 h compared to the virus control in each period, respectively. Similarly, algal polysaccharide extract reduced the plaque number of HSV-2 by 0.81, 1.03, 1.75, 2.14, and 3.01 log PFU mL^−1^ when treated at 0, 1, 2, 3, and 4 h compared to the virus control in each period, respectively. Moreover, algal polysaccharide extract at a concentration of 5000 μg mL^−1^ was also tested for the repression of HSV replication. The replication of HSV was carried out at 0, 6, 12, 18, 24, 30, and 36 h after treatment with algal polysaccharide extract, and was then compared to the virus control and ACV. ACV had 50% inhibitory concentration (IC_50_) values of 1.54 and 12.75 μg mL^−1^ for HSV‐1 and HSV‐2 treatment, respectively. These experimental conditions allow for the synchronization of the HSV replication steps. The results reveal that the extracellular HSV-1 and HSV-2 yields were inhibited 6 h after the infected cells were treated with algal polysaccharide extract. In addition, the extract also proved to repress the intracellular HSV yield. The inhibition trend of the intracellular HSV yield of HSV-1 and HSV-2 replication was greater than the action of ACV (Figs. 8 and 9).

## Discussion

Extract derived from natural substances provides effective treatment for various diseases. Polysaccharide extract from natural substances has broad potential effects against various viruses, especially enveloped viruses^34^. In previous studies, polysaccharide extract has proven its antiviral potency against human immunodeficiency viruses, Japanese encephalitis virus, dengue virus, yellow fever virus, influenza virus, avian influenza virus, and measles virus^35–38^. Studies of investigating the antiherpetic effects of sulfated polysaccharides have been conducted using polysaccharides from various sources. Sulfated polysaccharide extracts from green algae such as *Monostroma nitidum*, *Caulerpa brachypus*, *C*. *okamurai*, *C*. *scapelliformis*, *Chaetomorpha crassa*, *Ch*. *spiralis*, *Codium adhaerens*, *Co*. *fragille*, and *Co*. *latum* exhibited potent anti-HSV-1 activities during viral attachment, with 50% inhibitory concentrations (IC_50_) ranging from 0.38 to 8.5 μg mL^−1^^39^. The sulfated polysaccharides from these green algae exerted higher anti-HSV-1 activity than polysaccharide extract from *Cladophora* spp., which had EC_50_ value of 15.17 μg mL^−1^ in this study.

Moreover, the sulfated polysaccharide extract obtained from brown algae (*Sargassum muticum*) using enzyme-assisted and hot water extraction methods demonstrated its effectiveness in protecting Vero cells against HSV-1 infection, with EC_50_ values ranging from 37.7 to 48.0 μg mL^−1^. Similarly, the sulfated polysaccharide extract obtained from brown algae exhibited significant anti-HSV-1 activity in a post-HSV infection assay, with EC_50_ values ranging from 1.2 to 2.4 μg mL^−1^^40^. However, algal polysaccharide extract from *Cladophora* spp. in this study inhibited HSV-1 infection with lower activity when treatment before and after viral attachment with EC_50_ values of 70.31 and 9.78 μg mL^−1^, respectively.

In addition, the fractionated polysaccharide extract from red algae (*Callophyllis variegata*) exhibited anti-HSV-1 activities during viral attachment, with IC_50_ values ranging from 0.16 to 1.55 μg mL^−1^, and demonstrated anti-HSV-2 activities, with IC_50_ values ranging from 0.21 to 2.19 μg mL^−1^^41^. Moreover, the polysaccharide derived from *Spirulina platensis* (calcium spirulan) displayed potent anti-HSV-1 activity during viral attachment with an EC_50_ of 0.86 μg mL^−1^^42^. Thus, the polysaccharide from *Callophyllis variegata* and calcium spirulan polysaccharide from *Spirulina platensis* demonstrated higher activity than polysaccharide extract from *Cladophora* spp. in this study since the polysaccharide extract from *Cladophora* spp. inhibited HSV-1 and HSV-2 infection during viral attachment with EC_50_ values of 15.17 and 2.57 μg mL^−1^, respectively.

The study on polysaccharide extracts from seaweed including *Rhodymenia pseudopalmata*, *Solieria filiformis*, *Hydropuntia cornea* (Rhodophyta) and *Sargassum fluitans* (Phaeophyceae) was performed against HSV-1 infection. The result showed that all polysaccharide extracts had low toxicity on Vero cell with CC_50_ more than 200 μg mL^−1^. The polysaccharide extract from *Solieria filiformis* exhibited effective antiviral effect when treatment before viral attachment with EC_50_ of 136.0 μg mL^−1^ and SI value of 1.47 whereas the polysaccharide extract of *Sargassum fluitans* demonstrated antiviral activity with EC_50_ of 42.8 μg mL^−1^ and SI value of 4.67. The anti-HSV-1 activity of the algal polysaccharide extract from *Cladophora* spp. in this study was compared and the inhibition of HSV-1 infection before viral attachment (EC_50_ = 70.31, SI > 71.11) demonstrated higher anti-HSV-1 activity than the polysaccharide extract from *Solieria filiformis*. However, the polysaccharide extract of *Sargassum fluitans* showed stronger anti-HSV-1 activity than the polysaccharide extract from *Cladophora* spp. In contrast, *Rhodymenia pseudopalmata* and *Hydropuntia cornea* polysaccharide extracts did not show the anti-HSV-1 activity^43^.

Other study demonstrated anti-HSV activities of natural sulfated polysaccharides (SPs) from green algae; *Enteromorpha compressa* and *Monostroma nitidum*. The polysaccharide extracts showed low toxicity on Vero cell with CC_50_ values > 1000 and 4100 μg mL^−1^, respectively. The sulphated polysaccharides also exerted high efficacy of antiviral activity against HSV-1 infection during viral adsorption with the EC_50_ values of 0.49 and 0.4 μg mL^−1^ and the SI values > 200 and 1000, respectively. Moreover, the sulfated polysaccharide isolated from *Caulerpa brachypus*, *Caulerpa okamurai*, and *Caulerpa scapelliformis* also demonstrated low toxicity on Vero cell with CC_50_ values of 4700, 6400, and 6400 μg mL^−1^, respectively. This sulphated polysaccharide could inhibit HSV-1 during viral attachment with the EC_50_ of 1.9, 0.65, 0.55 μg mL^−1^ and SI values of 2500, 9800, and 12,000, respectively^39^. Therefore, the sulfated polysaccharide isolated from green algae showed the high potency of anti-HSV activity greater than the algal polysaccharide extract from *Cladophora* spp. that observed from this study (EC_50_ = 15.17, SI > 329.60).

Furthermore, a recent study revealed that marine polysaccharide extracts, such as sulfated polysaccharide extract from sea cucumber, fucoidan extract from brown algae, and ι-carrageenan extract from red algae, were able to inhibit SARS-CoV-2 infection on Vero E6 cells^44^. Other marine polysaccharides such as polysaccharide extracts from brown marine algae; *Undaria pinnatifida* sporophyll, *Laminaria japonica*, *Hizikia fusiforme*, and *Sargassum horneri* and green marine algae (*Codium fragile*), have demonstrated inhibitory activity against SARS-CoV-2 virus entry using the test model of SARS-CoV-2 pseudo-virus infection in ACE-2 overexpressed HEK293T cells^45^. In the same manner, sulfated polysaccharide extracts from algae have also demonstrated various effective biological activities, including immunomodulation, antiviral, antioxidant, antihyperlipidemic and anticancer activity^46^.

Hence, this study focused on algal polysaccharide extract from *Cladophora* spp. The algal polysaccharide extract from *Cladophora* spp. contains sulfated polysaccharides with high levels of carbohydrates and a very low protein and sulfate content. However, when the extraction involves a purification step, such as ion exchange chromatography, it could eliminate proteins and other organic compounds. Algal polysaccharide extract from *Cladophora glomerata* Kützing has been extracted with a purification step using DEAE-Sepharose fast flow ion exchange chromatography column, resulting in a reduced protein level from 17.3 to 13.0% w w^−1^. This purification step also increased the sulfate content of algal polysaccharide extract from 19.9 to 23.5% w w^−1^^47^. The FT-IR spectra shows the characteristics of algal polysaccharide extracts related to other algal polysaccharide extracts from an algal sample in the same order of Cladophorales, such as *Cladophora glomerata*, *Cladophora crispate*, *Cladophora surera*, *Cladophora rupestris*, *Chaetomorpha gracilis* Kützing, or *Rhizoclonium hieroglyphicum* (C.Agardh) Kützing^48–52^. The structural characteristics demonstrate the main functional group of the C–O–C bridge of the glycosidic linkage, and the sulfate group. Moreover, algal polysaccharide extract contains the functional group C=O of carboxylated sugars, which is similar to an analysis of sulfated glucan and sulfated galactan in a previous study. In accordance with this study, sulfated glucan and sulfated galactan are the main composition of the cell wall of the polysaccharide of *Cladophora* spp.^53^.

Algal polysaccharide extract from *Cladophora* spp. has a low toxicity on various cell lines, such as HGF-1 cells, RAW264.7 cells, and Vero cells. The results of this study are concordant with previous studies that evaluated the cytotoxicity of other algal polysaccharide extracts in cell culture, such as *Cladophora* spp., *Codium tomentosum*, *Ulva armoricana*, *Ulva intestinalis*, *Ulva lactuca*, and *Ulva pertusa*^54–58^. Furthermore, *Cladophora* spp. is proved to be experimentally safe by acute cytotoxicity tests on rats^59^.

Algal polysaccharide extract from *Cladophora* spp. shows high potential against HSV infection in Vero cells in a few different ways. The antiviral activity of algal polysaccharide extract against HSV prior and during viral adsorption on Vero cells is attributed to the protective and interfering properties of extract. Before viral adsorption, treatment of algal polysaccharide extract protects Vero cells by inhibiting HSV and during viral adsorption, the extracts interfere the viral infection step and blocks infection in Vero cells. Effect of algal polysaccharide extract on the single cycle of HSV-2 infection was determined by removal of the algal polysaccharide extract after viral adsorption. The efficacy of the algal polysaccharide extract after viral adsorption was rather low when the extract was removed by washing with phosphate-buffered saline after incubation for 1 h. This might be from the large molecular weight of algal polysaccharide extract that could not pass through the cell via cell membrane to inactivate the viral particles^60^. However, treatment of algal polysaccharide extract after viral adsorption without removal of the extract resulted in high efficacy of HSV inhibition since new viral particles might have time to expose to algal polysaccharide extract and the new viral particles were inactivated after viral egress from the infected cells.

The effectiveness of algal polysaccharides in inhibiting HSV-1 and HSV-2 infections on Vero cells varies depending on the infectious mechanism of each type of HSV. HSV infections involve viral glycoproteins binding to host cell receptors during viral fusion with the cytoplasmic membrane of host cell. The main receptors for cell entry are nectin-1, nectin-2, herpesvirus entry mediator (HVEM), and 3-O heparan sulfate (3-O HS), with HVEM and nectin-1 being used by both HSV-1 and HSV-2. However, 3-O HS is specific to HSV-1, while nectin-2 has a greater effect on HSV-2 entry than on HSV-1^61,62^. This suggests that algal polysaccharide extracts with polymer structures containing sulfate groups can interfere with the binding of viral glycoproteins to host cell receptors. Consequently, the algal polysaccharide extract has effect on anti-HSV infection during viral adsorption in Vero cells greater than other infection mechanisms. Moreover, similar results were obtained when cells were treated against enveloped viruses with green algal polysaccharides, sulfated polysaccharides and ulvan^39,63^.

Sulfated polysaccharides demonstrated antiviral activity against a wide range of viruses due to their unique polyanion structure, which carried a strong negative charge^64^. This characteristic enables them to inhibit viruses by interacting with the positive charges on the surface of host cells, thereby preventing virus penetration^65^. For example, sulfated polysaccharides can disrupt the viral glycoprotein region on viral particles by binding to the heparan sulfate proteoglycan region of the host cell surface^66^. These interactions correspond to the results of antiviral activity of algal polysaccharide extract against HSV both before and during viral adsorption on Vero cells. Moreover, the results of antiviral activity assays on algal polysaccharides against herpes simplex viruses indicate that sulfated polysaccharides can also disrupt the process of viral egress from host cells after viral adsorption and replication. Therefore, it would be beneficial to investigate and utilize natural compounds derived from *Cladophora* spp. algal extract that act in accordance with synthetic antiviral drugs on different targets of the HSV infection process.

## Materials and methods

### Cell line and viruses

In the cytotoxicity test, the human gingival fibroblasts cell line (HGF-1 cell; ATCC-CRL-2014) was used to represent a normal human primary cell, and the murine macrophage cell line originated from *Mus musculus* (RAW264.7; ATCC-TIB-71) was used to represent an immune cell. The African green monkey kidney cells (Vero cells) were kindly obtained from the Microbiology Section, Department of Medical Technology, Faculty of Associated Medical Science, Chiang Mai University, Chiang Mai, Thailand and was used as a model to study viral infections. All of the cell lines were cultured as a monolayer in a growth medium, Dulbecco’s modified Eagle medium; DMEM (Gibco, UK) supplemented with 10% v v^−1^ heat inactivated fetal bovine serum, FBS (Gibco, UK), 100 ug mL^−1^ streptomycin and 100 U mL^−1^ penicillin (Gibco, UK), 10 mM of 4-(2-hydroxyethyl)-1-piperazineethanesulfonic acid (HEPES). The cell was grown in a humidified 5% v v^−1^ CO_2_ atmosphere at 37 °C using a CO_2_ incubator until 80–90% confluence was observed. The standard strains of herpes simplex virus type 1 strain F (HSV-1F) and herpes simplex virus type 2 strain G (HSV-2G) were propagated on Vero cells cultured in DMEM medium containing 2% v v^−1^ FBS using a multiplicity of infection (MOI) of 1.0. The viral culture supernatant was collected to obtain the virus, and the titers of virus were quantified by plaque titration assay.

## Algal polysaccharide extraction

The specimen of *Cladophora* spp. was collected from the Mekong River, Wiang Sub-District, Chiang Khong District, Chiang Rai Province, Thailand and kindly verified by the applied algal research laboratory (AARL), Department of Biology, Faculty of Science, Chiang Mai University, Chiang Mai, Thailand. The *Cladophora* spp. specimen was dried at 60 °C and blended into a powder. The algal polysaccharides were extracted using a hot water extraction method. The dried *Cladophora* spp. was boiled in distilled water at 98 °C for 1 h with the ratio of 20 g algal powder to 1 L distilled water. Next, the extract solution was filtered and concentrated by a rotary evaporator. The extract was precipitated by 95% ethanol at 4 °C for 24 h. After precipitation, the precipitate was centrifugated and lyophilized to eliminate the ethanol from the extract^67^.

### Algal polysaccharide chemical profile analysis

The *Cladophora* spp. algal polysaccharide extract was analyzed for carbohydrate, protein, and sulfate content by colorimetric analysis technique. The total carbohydrate content was measured by phenol–sulfuric acid assay using D-glucose as a standard^68^. The algal polysaccharide extract was mixed with 5% w v^−1^ phenol solution. Thereafter, 98% w w^−1^ sulfuric acid was carefully added and the mixture was incubated for 10 min in the dark. After incubation, the reaction absorbance was measured at 490 nm by spectrophotometry (Thermo Scientific, USA). The protein content was determined by Lowry assay using bovine serum albumin as a standard^69^. The algal polysaccharide extract was complexed with Lowry's protein complex-forming reagent. After complexing, the reaction was mixed with 50% v v^−1^ Folin-Ciocalteu's phenol reagent, and was incubated for 30 min in the dark. The reaction absorbance was detected at 750 nm by spectrophotometry. Hydrolysis of the polysaccharides was performed with 4 M of hydrochloric acid at 100 °C for 2 h, according to the sulfate turbidity method using potassium sulfate as a standard, followed by an estimation of the sulfate content^70^. The digested algal polysaccharides were reacted with a sulfate turbidity conditioning reagent. The reaction was then gently mixed with 6% w v^−1^ barium chloride and the reaction absorbance was immediately measured at 420 nm by spectrophotometry.

### Algal polysaccharide structure characterization

The functional groups of sulphated polysaccharides of *Cladophora* spp. were analyzed by Fourier-transform infrared (FT-IR) spectroscopy using a Nicolet 6700 FT-IR spectrometer (Thermo Scientific, USA). The fully dried algal polysaccharide extract was embedded in potassium bromide (KBr) by the potassium bromide-pellet technique. The pellet was scanned in a wave number range of 4000–400 cm^−1^ with a resolution of 4 cm^−1^ using transmittance mode^71^.

### Cytotoxicity test of algal polysaccharides

The cytotoxic effects of algal polysaccharides from *Cladophora* spp. were determined on HGF-1 cells and RAW264.7 cells by 3-(4,5-dimethylthiazol-2-yl)-2,5-diphenyl-tetrazolium bromide (MTT) assay^72^. The cytotoxicity was also tested on Vero cells to determine a concentration of extract that could be used in subsequent antiviral studies. The algal polysaccharide extract at a maximal concentration of 5000 µg mL^−1^ was prepared in DMEM growth medium. The cultured cells were exposed to different concentrations of the extract, and the treated cells were incubated at 37 °C in a CO_2_ incubator for 48 h. After incubation, the MTT assay was prepared following the manufacturer’s instructions. A 5 mg mL^−1^ of MTT reagent (Bio Basic Inc., Canada) was added to each well and incubated at 37 °C for 4 h. Then, MTT-formazan from the treated cells was dissolved by dimethyl sulfoxide and the color of the formazan solution was determined using microplate readers (Biochrom, UK) by measuring the absorbance at 540 nm, with a reference wavelength of 630 nm. The cell viability percentage was calculated from the ratio between the absorbance values of both the treated cells and the untreated cells^73^.

### Antiviral assay of algal polysaccharides against herpes simplex viruses

The antiviral activities of *Cladophora* spp. algal polysaccharides were observed by plaque reduction assay with antiviral mechanisms, including the antiviral activity upon treatment before, during and after viral adsorption^74^.

The antiviral activity upon treatment before viral adsorption was evaluated. The Vero cells were seeded in a 24-well plate at a density of 1.0 × 10^5^ cells well^−1^. After incubation for 24 h, the cultured cell monolayer was treated with a non-toxic concentration of algal polysaccharide extract. The treatments were incubated at room temperature for 1 h. Then, the algal polysaccharide extract was disposed and the treated cells were washed twice with phosphate-buffered saline solution. The cells were then infected with HSV at the titers of 1.0 × 10^2^ PFU mL^−1^. The overlay medium containing 0.5% w v^−1^ carboxymethyl cellulose in growth medium was added to the wells for viral plaque forming. The infected cells were then incubated at 37 °C in a CO_2_ incubator for 72 h.

The antiviral activity upon treatment during viral adsorption was evaluated. The Vero cells were seeded in a 24-well plate at a density of 1.0 × 10^5^ cells well^−1^. After incubation for 24 h, a non-toxic concentration of algal polysaccharide extract and HSV at the titers of 1.0 × 10^2^ PFU mL^−1^ were inoculated onto the cell monolayer. The mixture was incubated at room temperature for 1 h. After incubation, the mixture was removed and the infected cells were washed twice with phosphate-buffered saline solution. The overlay medium containing 0.5% w v^−1^ carboxymethyl cellulose in growth medium was added to the wells for viral plaque forming. The infected cells were then incubated at 37 °C in a CO_2_ incubator for 72 h.

The antiviral activity upon treatment after viral adsorption was evaluated. The Vero cells were seeded in a 24-well plate at a density of 1.0 × 10^5^ cells well^−1^. After incubation for 24 h, the cultured cell monolayer was infected with HSV at the titers of 1.0 × 10^2^ PFU mL^−1^. The infected cells were incubated at room temperature for 1 h. The residual inoculum was then eliminated and the infected cells were washed twice with phosphate-buffered saline solution after the infected cells were treated with a non-toxic concentration of algal polysaccharide extract in the first group. Then, DMEM medium was added and further incubation during the remaining time up to 72 h. Whereas, the infected cells were not washed after treatment with the algal polysaccharide in the second group. The overlay medium containing 0.5% w v^−1^ carboxymethyl cellulose in growth medium was added to the wells for viral plaque forming. The infected cells were then incubated at 37 °C in a CO_2_ incubator for 72 h.

After 72 h of incubation in all antiviral assays, the viral plaques were stained with 0.1% crystal violet in 0.5% ethanol. The viral plaque formation was counted and the percentage of relative plaque forming units was calculated from the amount of plaque forming units remaining from the inactive by the algal polysaccharide extract, related to the amount in the virus control. The viral inhibition efficiency was compared to acyclovir and heparin. Accordingly, the acyclovir drug, ACV (Sigma-Aldrich, USA), was used as the positive control for antiviral activity upon treatment after viral adsorption. On the contrary, the heparin sodium salt was used as the positive control of antiviral activity upon treatment before viral adsorption and antiviral activity upon treatment during viral adsorption. The 50% effective concentration (EC_50_) values of the extract against the virus were calculated. The selective index (SI) values were also analyzed from the ratio between the CC_50_ value and EC_50_ value^75^.

### Virucidal assay of algal polysaccharides on herpes simplex viruses

A viral suspension of HSV particles at the titers of 1.0 × 10^4^ PFU mL^−1^ was mixed with the equivalent volume of a non-toxic concentration of *Cladophora* spp. algal polysaccharide extract. The mixtures were incubated at room temperature for 1, 2, 3, and 4 h to allow the algal polysaccharide extract to have an effect on the virus particles. After incubation for the respective times, the residual virus was diluted 100 times using DMEM to eliminate the effects of the remaining algal polysaccharide extract on subsequent binding events. In this case, the titer of the virus and the concentration of the extract tested are 1.0 × 10^2^ PFU mL^−1^ and 50 µg mL^−1^, respectively. This latter concentration is close to, but not greater than, EC_50_, so it has no significant influence on virus inhibition. The diluted virus was infected into Vero cells and incubated at room temperature for 1 h. The HSV titer was determined by plaque titration assay^76^.

### Antiviral replication assay of algal polysaccharides against herpes simplex viruses

Vero cells were seeded in a 6-well plate at a density of 1 × 10^5^ cells well^−1^. After incubation for 24 h, the cultured cell monolayer was infected with HSV at an MOI of 0.1. The mixture was incubated at room temperature for 1 h. After incubation, the residual inoculum was removed and the infected cells were washed twice with phosphate-buffered saline solution. The infected cells were treated with non-toxic concentrations of *Cladophora* spp. algal polysaccharide extract and were incubated at 37 °C in a CO_2_ incubator. Next, the supernatants were collected at 0, 6, 12, 18, 24, 30, and 36 h after viral infection to harvest the extracellular virus. In a like manner, the infected cells were also frozen and thawed twice to harvest the intracellular virus. The harvested virus was kept at -80 °C before the determination of virus titers using plaque titration assay^77^.

### Statistical analysis

Statistical analyses were executed by IBM SPSS Statistics 20 software (IBM Corp., USA). A one-way analysis of variance (ANOVA) was performed and Tukey's honestly significant difference (HSD) post hoc test was used to establish the significance among all groups with the significant difference at *p*-values less than 0.05 (*p* < 0.05). The EC_50_ values were estimated using probit analysis by PriProbitNM 1.63 software (Kyoto University, Japan)^78^.

## Conclusions

The characterization of *Cladophora* spp. by chemical analysis established that the structure of algal polysaccharide extract is sulfated polysaccharides. Algal polysaccharide extract exhibited low toxicity on HGF-1 cells, RAW264.7 cells, and Vero cells, and showed high anti-herpetic activity against both HSV-1 and HSV-2 infections upon treatment before and during viral adsorption on Vero cells. The algal polysaccharide extract also had high antiviral activity on both HSV-1 and HSV-2 when treatment after viral adsorption and leaving for 72 h of viral infection on Vero cells without removing the extract from the infected cells. Moreover, algal polysaccharide extract demonstrated direct inactivation of HSV viral particles in virucidal assay. Furthermore, algal polysaccharide extract showed greater inhibitory effect on HSV replication than the IC_50_ dose of the ACV drug.

## Data Availability

The data presented in this study are available on request from the corresponding author.
